# Social Cognition, the Male Brain and the Autism Spectrum

**DOI:** 10.1371/journal.pone.0049033

**Published:** 2012-12-26

**Authors:** Jeremy Hall, Ruth C. M. Philip, Katie Marwick, Heather C. Whalley, Liana Romaniuk, Andrew M. McIntosh, Isabel Santos, Reiner Sprengelmeyer, Eve C. Johnstone, Andrew C. Stanfield, Andy W. Young, Stephen M. Lawrie

**Affiliations:** 1 Division of Psychiatry, University of Edinburgh, Edinburgh, United Kingdom; 2 Department of Educational Sciences, University of Aveiro, Aviero, Portugal; 3 Department of Psychology, St Andrew's University, St Andrew's, United Kingdom; 4 York Neuroimaging Centre, University of York, York, United Kingdom,; The University of Western Australia, Australia

## Abstract

Behavioral studies have shown that, at a population level, women perform better on tests of social cognition and empathy than men. Furthermore Autism Spectrum Disorders (ASDs), which are characterized by impairments in social functioning and empathy, occur more commonly in males than females. These findings have led to the hypothesis that differences in the functioning of the social brain between males and females contribute to the greater vulnerability of males to ASD and the suggestion that ASD may represent an extreme form of the male brain. Here we sought to investigate this hypothesis by determining: (i) whether males and females differ in social brain function, and (ii) whether any sex differences in social brain function are exaggerated in individuals with ASD. Using fMRI we show that males and females differ markedly in social brain function when making social decisions from faces (compared to simple sex judgements) especially when making decisions of an affective nature, with the greatest sex differences in social brain activation being in the inferior frontal cortex (IFC). We also demonstrate that this difference is exaggerated in individuals with ASD, who show an extreme male pattern of IFC function. These results show that males and females differ significantly in social brain function and support the view that sex differences in the social brain contribute to the greater vulnerability of males to ASDs.

## Introduction

Previous behavioural studies have suggested that women perform better in tests of social cognition and empathy than males [1]–[4], although these findings have not been without controversy. Studies using questionnaires have shown that women score more highly than men on measures of empathy and social function [1], [2], [5], but such self-report measures may be influenced by differential motivation rather than true biological differences in social performance. Experimental evidence of sex differences in social function has been obtained from studies looking at facial emotion expression processing which have suggested a female advantage in decoding emotional expressions from faces and from the eye region alone [3], [4], [6], [7]. However sex differences in facial emotion processing are generally subtle and restricted to low-intensity emotions [8].

Imaging studies have provided additional evidence of sexual dimorphism in regions which contribute to social functioning. Longitudinal structural imaging studies have demonstrated sex differences in the trajectory of brain development, with females showing earlier overall cerebral development and greater relative frontal lobe grey matter volumes [9]. Cross-sectional studies in adults have confirmed that females have larger relative volumes of a number of brain regions implicated in social function including the inferior frontal cortex (IFC) [10]–[14], cingulate cortex [12], [15] and inferior parietal cortex [12], [15], [16], whilst men have larger relative volumes of the amygdala [12], [17] and cerebellum [12], [15]. There have been fewer functional imaging studies that have investigated sex differences in the social brain but investigations of face processing have suggested a degree of sexual dimorphism in brain function which depends in part on the nature of the stimuli used [18]–[22].

Sexual variation in social brain regions is potentially of relevance to autism spectrum disorder (ASDs) which are characterised by impairments in social cognition and are at least four times more common in males than females [23]. People with ASD score lower on questionnaire measures of empathy than healthy males (who in turn score lower than healthy females) [1], and perform relatively poorly on tests of emotion recognition and social judgement [24], [25]. Furthermore individuals with ASD have been found in some studies to display an exaggerated male pattern of neuroanatomy [26], [27] and there is evidence from functional imaging studies that individuals with ASD may exhibit a male pattern of brain activation in regions including the IFC [22], [28].

In view of existing evidence that men and women's social functioning may differ at a neural level, and that those with ASD may have an exaggerated male pattern of social brain function, in the present study we sought to determine whether males and females differ in terms of social brain function while viewing faces during fMRI, and to relate these findings to brain activation in individuals with ASD.

## Results

### Male and female brain activation during social judgement

We compared male and female brain activation during social judgement in forty seven volunteers (25 males and 22 females). All participants completed two social judgement tasks during the fMRI session. In the first task participants were asked to rate whether faces appeared approachable or not, a primarily affective social decision. In the second task participants rated faces according to whether they appeared intelligent or not, a primarily cognitive social decision [29]. A matched perceptual control condition was used for both tasks, which consisted of determining male or female sex from the same faces, counterbalanced across participants. There were no difference between the sexes in terms of accuracy of performance in wither the approachability task (gender judgements F_1,45_ = 0.40, P>0.5; approachability judgements F_1,45_ = 0.50, P>0.4) or intelligence task (gender judgements F_1,45_ = 0.02, P>0.8; intelligence judgements F_1,45_ = 0.04, P>0.8) (Table 1). In terms of reaction times male participants were slightly, but significantly, slower than females in making social judgements of both approachability (F_1,45_ = 4.24, P = 0.046) and intelligence (F_1,45_ = 4.75, P = 0.035) from faces (Table 1). There was however no difference between the sexes in reaction times for sex judgements during either task (P>0.2 in all cases) (Table 1).

**Table 1:** Behavioural performance in the approachability and intelligence judgement tasks.

Approachability Judgement Task				
***Comparison of males and females***				
	Sex Accuracy	App Accuracy	Sex RT	App RT
Females	96.0 (6.1)	90.9 (10.1)	1067 (206)	1285 (237)
Males	95.0 (4.7)	88.7 (10.7)	1124 (157)	1447 (289)
***Comparison of males with ASD to control males***				
	Sex Accuracy	App Accuracy	Sex RT	App RT
Control Males	94.7 (4.0)	88.2 (11.2)	1151 (275)	1455 (365)
ASD Males	93.3 (3.8)	79.9 (15.7)	1241 (220)	1558 (307)

Intelligence Judgement Task				
***Comparison of males and females***				
	Sex Accuracy	Int Accuracy	Sex RT	Int RT
Females	95.4 (10.7)	79.8 (12.9)	1029 (231)	1468 (283)
Males	95.8 (4.0)	79.0 (11.5)	1073 (149)	1658 (303)

Accuracy is shown as percentage correct responses. Reaction time is given in milliseconds. Standard deviations are given in parentheses. App = approachability judgements Int = intelligence judgements; Sex = sex discrimination judgements.

**Figure 1:**
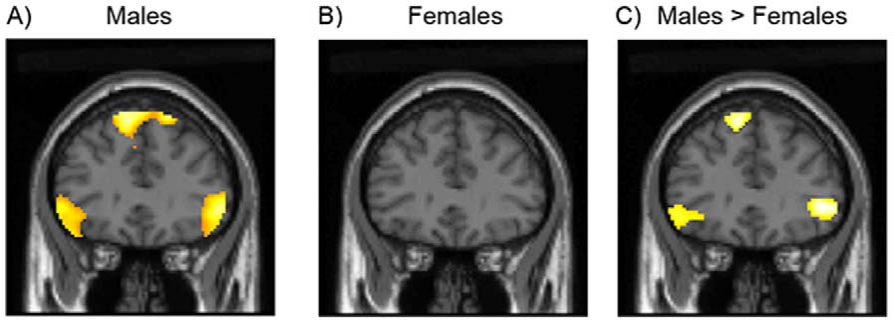
Brain activation during approachability judgements in A) males and B) females (SPM thresholded P<0.001). C) Between group comparison showing greater activation of inferior frontal cortex (IFC) in males than in females during approachability judgements (SPM thresholded P<0.005).

**Figure 2:**
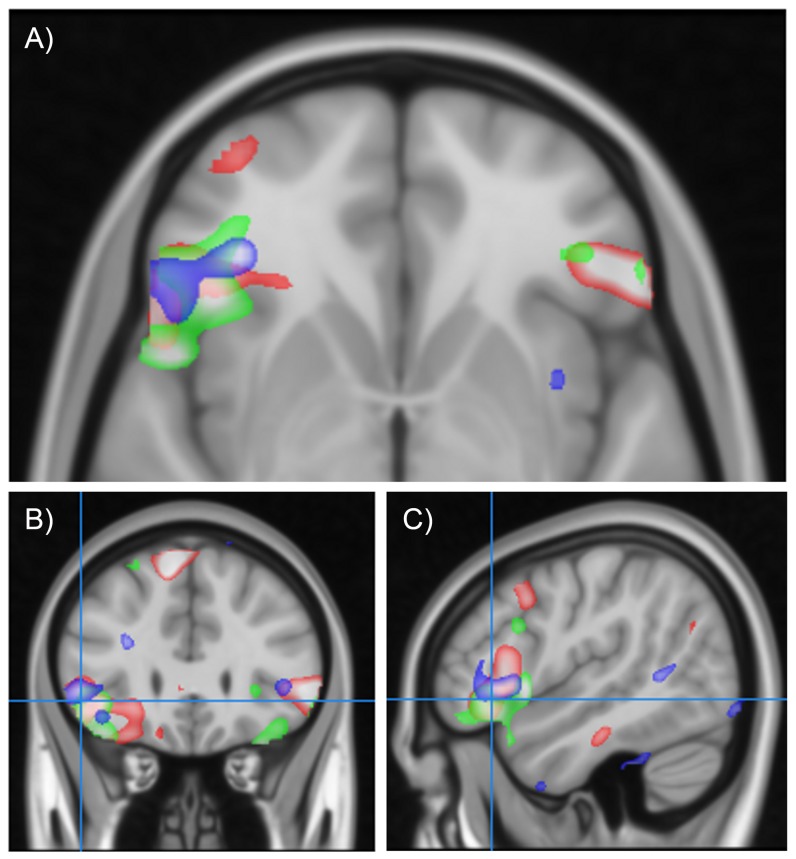
Convergent activation changes in the left inferior frontal cortex in males and individuals with ASD. Red scale indicates regions showing greater activation in males than females. Blue scale indicates regions showing greater activation in participants with ASD than controls. Green scale indicates regions correlating with empathy in males. A) Transverse view, B) Coronal view, C) Saggital view. All contrasts thresholded at T>2.5.

Analysis of the fMRI data revealed marked differences in the function of male and female brains during affective social decision-making. Male participants showed increased brain activation when making social judgements of approachability compared to simple judgements of sex, with activation differences localized to regions of the social brain including the medial prefrontal and bilateral inferior frontal cortices (Figure 1A and Table 2). However, strikingly, female participants did not show this increase in brain activation when making approachability judgements compared to perceptual judgements of the face's sex (Figure 1B and Table 2). Direct comparison of brain activation in males and females revealed a significant difference between the sexes in the left inferior frontal cortex (IFC) (Peak −50, 22, 8; total extent 678 voxels; T = 3.56; cluster Pcorr = 0.034; Figure 1C, Figure 2 and Table 2), with males showing greater left IFC activation. Males and females also showed a different relationship between social brain activation and empathy, with males showing a positive whole brain correlation between left IFC activation and empathy scores (EQ) during approachability judgements (Peak −52, 12, −2; extent 743 voxels; T = 4.03; cluster Pcorr = 0.022; Figure 2) which was not seen in females. Men and women did not differ in brain activation when making judgements of intelligence from faces, suggesting that the sex differences in social brain function are not seen in social decisions that are less related to appraisal of affective state and potential threat (Table 3) [29]. These findings demonstrate that men and women differ in social brain function with particularly marked differences between the sexes evident in the left IFC during judgements of approachability.

**Table 2:** Regional brain activation during the approachability task for males and females (contrast of approachability judgements versus gender judgements).

Approachability Task (Approachability Judgements versus Sex Judgements)				
Cluster P	Extent	T	Peak voxel	Region
***Males within group activation***				
<0.001	3855	7.35	−10 28 58	Medial Prefrontal Cortex
<0.001	839	6.75	54 28 −2	R Inferior Frontal Cortex
<0.001	1980	6.44	−32 −88 −36	L Cerebellum
<0.001	1952	6.08	−54 20 −6	L Inferior Frontal Cortex
<0.001	1029	5.79	26 −90 −36	R Cerebellum
***Females within group activation***				
No significant clusters				
***Between Group Contrast (Males>Females)***				
0.034	678	3.56	−50 22 0	L Inferior Frontal Cortex
***Between Group Contrast (Females>Males)***				
No significant clusters				

SPMs thresholded at P<0.001 for within group contrasts and P<0.005 for between group contrasts.

**Table 3:** Regional brain activation during the intelligence task for males and females (contrast of intelligence judgements versus gender judgements).

Intelligence Task (Intelligence Judgements versus Sex Judgements)				
Cluster P	Extent	T	Peak voxel	Region
***Males within group activation***				
<0.001	16458	9.06	−46 22 −14	L Inferior Frontal Cortex extending to Medial Prefrontal Cortex
<0.001	6737	8.84	−30 −86 −42	Cerebellum
<0.001	1835	7.27	46 12 46	R Dorso-lateral Prefrontal Cortex
<0.001	1210	6.76	48 26 −16	R Inferior Frontal Cortex
0.001	467	5.83	−60 −30 −8	L Temporal Cortex
***Females within group activation***				
<0.001	9757	9.31	−42 12 36	L Inferior Frontal Cortex
<0.001	4227	9.27	−40 −74 −46	Cerebellum
<0.001	7254	7.37	10 58 28	Medial Prefrontal Cortex
0.009	275	6.53	32 44 −16	R Inferior Frontal Cortex
***Between Group Contrast (Males>Females)***				
No significant clusters				
***Between Group Contrast (Females>Males)***				
No significant clusters				

SPMs thresholded at P<0.001 for within group contrasts and P<0.005 for between group contrasts.

We further investigated whether there were any differences between males and females in brain activation during social judgements or judgements of sex from faces compared to the baseline rest condition could account for these sex differences (Tables 4–7). There were no differences between males and females in brain activation in the IFC region when social/sex judgements were compared to the baseline rest condition, indicating that the sex differences in IFC activation did not arise from differences in response to the sex-judgement comparison condition (Tables 4–7). It is however interesting to note that females showed bilaterally greater activation of the insula when making simple sex judgements from faces (compared to baseline rest) in the approachability task, suggesting that females do process facial information differently to males even when not explicitly making higher-order social judgements (Tables 4 and 5). These differences in activation between sexes were however in a different region from those seen in the more constrained contrast of approachability judgements versus sex judgements and therefore could not account for the observed differences in IFC activation seen between the sexes.

**Table 4:** Regional brain activation during the approachability task for males and females for sex judgements compared to the baseline rest condition.

Approachability Task: Sex Judgements versus Rest				
Cluster P	Extent	T	Peak voxel	Region (peak)
***Males within group activation***				
<0.001	5536	10.96	36 −48 −28	R Cerebellum
<0.001	5420	9.02	−16 −104 −4	L Cuneus
<0.001	3816	8.13	46 6 32	R Inferior Frontal Gyrus
<0.001	4901	7.74	−46 −24 62	L Postcentral Gyrus
0.011	356	6.61	36 44 −18	R Inferior Frontal Cortex (BA11)
0.003	465	5.52	32 −60 50	R Superior Parietal Lobule
0.015	332	5.42	−34 26 4	L Inferior Frontal Gyrus
0.000	820	5.26	−38 −4 14	L Insula
***Females within group activation***				
<0.001	18005	11.49	24 −92 −6	R Lingual Gyrus
<0.001	17396	8.36	−62 −2 42	L Precentral Gyrus
<0.001	1032	6.81	−10 −10 56	L Medial Frontal Gyrus
<0.001	1261	6.04	32 −58 46	R Superior Parietal Lobule
0.032	234	5.70	−56 2 42	L Middle Frontal Gyrus
***Between Group Contrast (Males>Females)***				
No significant clusters				
***Between Group Contrast (Females>Males)***				
0.011	882	5.07	−42 −6 −2	L Insula
0.004	1060	3.94	50 −30 6	R Insula and Superior Temporal Gyrus

SPMs thresholded at P<0.001 for within group contrasts and P<0.005 for between group contrasts.

**Table 5:** Regional brain activation during the approachability task for males and females for social judgements compared to the baseline rest condition.

Approachability Task: Approachability Judgements versus Rest				
Cluster P	Extent	T	Peak voxel	Region (peak)
***Males within group activation***				
<0.001	16458	10.17	−14 −104 −6	L Cuneus
<0.001	25958	9.41	34 24 −4	R Inferior Frontal Gyrus
<0.001	3242	7.08	2 −18 −14	Brainstem
***Females within group activation***				
<0.001	23480	11.45	38 −54 −22	R Cerebellum
<0.001	27113	10.83	26 48 −22	R Inferior Frontal Gyrus
<0.001	1814	8.80	44 −60 50	R Superior Parietal Lobule
***Between Group Contrast (Males>Females)***				
0.004	1019	5.09	−14 56 8	L Medial Frontal Gyrus
***Between Group Contrast (Females>Males)***				
0.003	1077	5.62	44 −8 2	R Insula
0.032	683	5.20	−44 −8 −2	L Insula

SPMs thresholded at P<0.001 for within group contrasts and P<0.005 for between group contrasts.

**Table 6:** Regional brain activation during the intelligence task for males and females for sex judgements compared to the baseline rest condition.

Intelligence Task: Sex Judgements versus Rest				
Cluster P	Extent	T	Peak voxel	Region
***Males within group activation***				
<0.001	11363	8.33	−30 −86 −14	L Inferior Occipital Gyrus
<0.001	2362	7.38	−60 −24 52	L Postcentral Gyrus
0.023	325	6.40	−42 −6 18	L Insula
<0.001	943	5.79	44 4 32	R Inferior Frontal Gyrus
0.002	531	5.45	10 66 −20	R Inferior Frontal Cortex (BA11)
0.041	280	4.72	−6 −4 58	R Medial Frontal Gyrus (BA6)
***Females within group activation***				
<0.001	14425	15.20	36 −52 −24	R Cerebellum
<0.001	5117	10.79	−54 −30 58	L Postcentral Gyrus
<0.001	788	8.72	26 54 −20	R Inferior Frontal Cortex (BA11)
<0.001	606	6.10	56 28 28	R Middle Frontal Gyrus (BA46)
<0.001	503	5.65	36 24 −2	R Inferior Frontal Gyrus
0.009	305	5.19	52 −36 44	R Inferior Parietal Lobule
0.023	247	4.85	−26 −24 −4	L Hippocampus
***Between Group Contrast (Males>Females)***				
No significant clusters				
***Between Group Contrast (Females>Males)***				
No significant clusters				

SPMs thresholded at P<0.001 for within group contrasts and P<0.005 for between group contrasts.

**Table 7:** Regional brain activation during the intelligence task for males and females for social judgements compared to the baseline rest condition.

Intelligence Task: Intelligence Judgements versus Rest				
Cluster P	Extent	T	Peak voxel	Region
***Males within group activation***				
<0.001	16111	9.56	20 −102 −6	R Lingual Gyrus
<0.001	4778	9.26	−40 24 −16	L Inferior Frontal Gyrus
<0.001	9573	8.42	6 30 68	R Superior Frontal Gyrus
<0.001	7268	7.02	50 10 56	R Middle Frontal Gyrus
<0.001	1537	6.12	−52 −24 62	L Postcentral Gyrus
0.005	461	5.59	2 −54 −32	R Cerebellum
***Females within group activation***				
<0.001	17105	12.81	−42 −64 −22	L Cerebellum
<0.001	15679	11.37	54 36 32	R Middle Frontal Gyrus
<0.001	4390	8.61	22 32 62	R Superior Frontal Gyrus
<0.001	1512	7.21	−52 −26 62	L Postcentral Gyrus
<0.001	730	6.59	48 −56 50	R Inferior Parietal Lobule
0.036	225	6.02	−38 54 28	L Superior Frontal Gyrus
***Between Group Contrast (Males>Females)***				
0.013	844	5.10	−8 14 −14	L Subcallosal Gyrus (BA25)
***Between Group Contrast (Females>Males)***				
No significant clusters				

SPMs thresholded at P<0.001 for within group contrasts and P<0.005 for between group contrasts.

### Social brain activation during social judgement in ASD

We next investigated brain activation during social judgement in Autism Spectrum Disorders (ASD). Twelve males with a diagnosis of ASD and twelve healthy male controls males participated in the study. Participants were matched in terms of age, handedness and IQ. Individuals from the ASD group showed significantly higher levels of autistic symptoms as assessed on the autism quotient (AQ) (P<0.001) [30], [31]. All participants completed the approachability task during fMRI scanning. There was no difference between the groups in terms of accuracy of task performance for either sex judgements or social judgements (F_1,22_ = 0.75, P>0.3 and F_1,22_ = 2.37, P>0.1 respectively) or in terms of reaction times for either gender or social judgements (F_1,22_ = 0.80, P>0.3 and F_1,22_ = 0.75, P>0.3 respectively) (Table 1). Individuals with ASD showed greater activation of the left IFC during approachability judgements (compared to judgements of sex) than healthy males (Peak −36, 36, −14 with secondary peak at −50, 20, 0; cluster extent 210 voxels; peak T = 4.32; cluster Pcorr = 0.049 within bilateral IFC mask; Table 8), paralleling the difference in IFC activation seen between healthy male and female participants (Figure 2).

**Table 8:** Comparison of brain activation during the approachability task between the ASD group and matched male control participants.

Approachability Task (Approachability Judgements versus Sex Judgements)				
Cluster P	Extent	T	Peak voxel	Region
***ASD>Controls***				
0.049*	210	4.32	−36 36 −14	L Inferior Frontal Cortex
***Controls>ASD***				
0.047	495	4.16	16 42 14	R Anterior Cingulate Cortex

SPMs thresholded at P<0.005.

## Discussion

In the present study we demonstrate sex differences in social brain function while making judgements of approachability from faces which are greatest in the left inferior frontal cortex (IFC). We further demonstrate that differences in activation in the IFC region differentiate men with ASD from neurotypical males. Notably these differences were evident despite matched accuracy of task performance, controlling for an important potential confound. Overall these studies demonstrate significant sexual dimorphism in social brain function which may contribute to the greater vulnerability of males to ASD.

The IFC is part of the social brain network and has been previously been shown to be activated when individuals make social judgements from faces [29], [32]. Lesion studies have found that damage to the IFC impairs emotional empathy but not cognitive empathy, as assessed by self-report questionnaires, facial expression identification tasks, and theory of mind tasks [33]. The IFC is considered part of the fronto-parietal mirror neuron system, which may have a role in understanding the mental states of others (for reviews see [34], [35]), although mirror neuron function was not explicitly tested in the present studies.

Sex differences in the IFC have been identified in both structural and functional imaging studies. Structural MRI studies have shown that women have larger regional grey matter volumes in a number of regions related to social information processing including the IFC [10]–[13], [36], [37], and have greater cortical folding in the IFC and parietal cortex [38]. Self-reported social co-operativeness has been found to correlate with volume of the posterior IFC bilaterally and the left anterior medial prefrontal cortex [10]. These findings were partially replicated by Cheng et al., 2009 [11] who found larger female grey matter volumes in the right posterior IFC (pars opercularis), right inferior parietal lobule and right medial prefrontal cortex, and that volumes in all these regions correlated with self-reported empathy.

Functional MRI studies have also provided evidence of sex differences in the IFC. fMRI studies have shown greater female activation of the right IFC during evaluation of facial emotion [39], of the left IFC during attribution of likely facial emotion [21] and of the IFC bilaterally during the “Reading the Mind in the Eyes” test [22]. Sex related activation differences may vary with social stimulus: men showed greater activation than women in the left IFC and associated brain regions when viewing contemptuous faces, but women showed greater activation in the left IFC when viewing disgusted faces [19]. Aleman and Swart [19] suggested this could be related to a greater male interest in social hierarchy. Possibly consistent with this, men were found to activate the right IFC more than women when viewing children's faces contrasted with adult faces [40].

Our finding of increased male left IFC activation when judging approachability may reflect the implicit requirement to assess potential threat and social dominance contained within an assessment of approachability. Alternatively, it may be that men are less efficient at making such affective judgements, and require greater regional blood flow for their completion. It is not however possible to fully elucidate the explanation for the differences in IFC activation between males and females within the current dataset. It is interesting to note that there was a positive correlation between empathy scores (as measured by the EQ) and IFC activation within males but not females. One potential interpretation of this finding would be empathic males have adapted to underlying differences in the social brain (especially the IFC) between males and females by showing greater recruitment of this brain region during social tasks. However full exploration of these possibilities would require further studies with parametric variation of both sex of participant and task difficulty.

The key finding of our second study is that men with ASD also show greater blood flow to the left IFC than neurotypical men while performing the same test of approachability from faces. This is consistent with previous work implicating the IFC in ASD. The IFC (pars opercularis) has been found to be smaller in ASD by voxel based morphometry [41], [42], manual tracing [43] and automated cortical thickness assessment [44]. IFC volume reductions in ASD have also been found to correlate with observer rated social impairment [43], [44].

Previous fMRI studies have also implicated the IFC in ASD. Reduced IFC activation has been reported in people with ASD performing the “Reading the Mind in the Eyes” test [28] and reduced right pars opercularis activity in children with ASD viewing facial emotions, which correlated with severity of observer rated social impairment [45]. In contrast, during irony comprehension children with ASD were found to show greater activation in the right IFC [46]. This may reflect the interaction of task difficulty with brain activation in dysfunctional brain regions, whereby easier tasks are associated with more activation than controls, and harder tasks with lesser activation. Such an effect has been observed for other frontal brain regions and tasks in other neuropsychiatric conditions. For example patients with schizophrenia show a leftward shift in the relationship between dorsolateral frontal activation and task load during working memory tasks [47].

It is striking that the left IFC, which showed the greatest activation difference between men and women during a test of social judgement, also showed a significant difference in activation between individuals with ASD and controls in the same experimental paradigm. These findings support the view that differences in social brain function between the sexes contribute to the higher rates of ASDs seen in males, potentially through convergent effects on the function of the IFC. The results are also consistent with the “extreme male brain theory” of autism which suggests ASDs are associated with an exaggeration of normal male vs female neural and psychological differences, possibly due to heightened exposure to prenatal androgens, or by the cumulative action of risk genes differentially expressed in males and females [24]. However the results are also compatible with a more general convergence of sex differences in social brain function with neural risk for ASD.

We note some limitations to the current study. Firstly, the task used tested only a restricted range of social cognitive function (the judgement of particular attributes from static images of faces) and therefore the broader generalisability of these findings needs to be determined in further studies. Secondly, we did not include females with ASD and therefore we cannot draw conclusions about social brain function in this important (although less common) group.

In summary, the present results demonstrate a marked difference in social brain function between men and women which is accentuated in individuals with ASD, suggesting a neurobiological substrate for the increased rates of ASDs in males.

## Materials and Methods

### Ethical approval

All participants gave written informed consent approved by the Lothian Research Ethics Committee. Participants were able to withdraw from the study at any stage. Participants who withdrew from the study remained eligible for all treatments (where required) and were not disadvantaged in any way.

### Participants and behavioural measures

Forty-seven healthy control participants were recruited (25 males and 22 females) for the first study comparing social brain activation in males and females. The groups were well matched in terms of age (males mean 32.7 years (SD = 8.1); females mean 31.7 years (SD = 9.0)), years of education (males mean 16.6 years (SD = 2.1); females mean 16.8 years (SD = 1.8)) and NART IQ (males mean 117.9 (SD = 6.4); females mean 116.1 (SD = 6.0)) (P>0.3 for all). All participants were right handed. Exclusion criteria included a history of neurological or psychiatric disorder, substance dependence and factors precluding MRI scanning. All participants completed questionnaire measures of empathy and systematizing using the using the empathy quotient (EQ) and systematizing quotient (SQ). Mean scores by gender were: males EQ 45.3 (SD = 9.9); SQ 34.9 (SD = 10.0); females EQ 50.4 (SD = 8.2); SQ 21.6 (SD = 9.7).

For the second study investigating the social brain activation in ASD we recruited 12 new male healthy control participants and 12 male individuals with ASD. One individual with ASD was excluded from analysis due to scanner artefacts. Additional exclusion criteria were as above. Groups were matched in terms of age (ASD group mean 36.6 (SD 12.0), control group mean 35.1 (sd 10.3)) and IQ (ASD group mean 105.8 (SD 20.8), control group mean 109.3 (sd 10.5)). All subjects were right handed. All individuals in the ASD group were interviewed by a clinician and case notes were reviewed to confirm a DSM-IV diagnosis of Asperger's syndrome (8 individuals) or high functioning autism (4 individuals). Autistic symptoms were rated in all participants using the Autism Quotient (AQ). AQ scores in the ASD group were mean 33.5 (SD 7.0) and in the control group were mean 14.3 (SD 3.8). In addition both the ASD group and the control group also completed the EQ (mean scores: 33.5 (SD 8.3) and 50.5 (SD 11.4) respectively) and SQ questionnaires (mean scores: 32.1 (SD 17.8) and 32.6 (SD 9.8) respectively).

### Task Design

The approachability and intelligence judgement tasks were performed as described previously [29]. In the approachability task, participants had to decide whether faces appeared ‘not approachable’ or ‘very approachable’. In the intelligence task, participants had to decide whether the faces appeared ‘not intelligent’ or ‘very intelligent’. Faces were selected from a large battery of 1000 face images to represent the extremes of each social dimension.

The stimuli were colour photographs of Caucasian male and female adult faces selected from a previously collected database of 1000 photographs of faces of non-famous people [48]. All the pictures were cropped around the face and hair, so that the minimum possible clothing and background were visible. The photographs were all adjusted to the same height (150 pixels; approximately 5 cm on the screen display), while the width varied slightly. No other attempt was made to standardise the pictures. Instead, the database included photographs that covered a wide range of adult ages, poses, and expressions, so that as many as possible of the naturally occurring cues would be present in the images. All the photographs had been rated on several characteristics with 1 to 7 point scales for all characteristics. The faces were highly reliably rated on both social dimensions across all raters (P<0.01; Cronbach's alpha 0.79 for approachability judgements and 0.75 for intelligence judgements). Faces representing the extremes of each social dimension were selected as stimuli for the neuroimaging task. There was a low overall correlation (0.26) between decisions made on the approachability and intelligence judgement tasks, suggesting that these tasks test different dimensions of social judgement [48].

Two sets of facial stimuli (A and B) were assembled for each task. The sets consisted of 18 male and 18 female faces each. The faces of each sex were selected to maximise the difference across each social dimension examined (for example, in the approachability condition, 9 high approachability faces and 9 low approachability faces of each gender per set). For each participant one set of faces was used for social judgements and the other set of faces was used for gender judgements. The use of the stimulus sets was counterbalanced across participants such that half the participants made social judgements from stimulus set A and control gender judgements from stimulus set B and half the participants made social judgements from stimulus set B and control gender judgements from stimulus set A [29].

Each task consisted of two runs of six 25 s blocks per run, with blocks of social judgements (“social” condition) alternating with blocks of gender judgements (“gender” condition). All blocks were separated by a 12.5 s rest period. Six faces were shown per block. The alternative response choices were shown on the screen (eg “not approachable” and “very approachable”) and participants had to press a button to indicate which response they felt was most appropriate for each face shown. Responses on the social judgement tests were scored according to their agreement with normative studies and reaction times were recorded for all participants [29], [48]. Task order was counterbalanced across participants.

### Image Acquisition and Analysis

Imaging was performed on GE 1.5TE Signa scanner (GE Medical). Functional scanning comprised 99 volumes per run (Field of View 22 cm; Time to Echo (TE) 40 ms; Volume acquisition time (TR) 2.5 s). Axial slices were acquired with a thickness of 5 mm and matrix size of 64×64. EPI images were reconstructed offline in ANALYZE format (Mayo Foundation, Rochester, MN, USA). Image processing was conducted using SPM2 (http://www.fil.ion.ucl.ac.uk/spm/software/spm2/). Pre-processing consisted of re-orientation of the images and realignment to the mean EPI image. Within-scanner movement was examined and an exclusion criterion of movement>3 mm over less than 20 consecutive images was applied. No subjects were excluded due to excessive movement. Images were subsequently normalised to the standard Montreal Neurological Institute EPI template and smoothed using a Gaussian kernel (8 mm^3^ full-width at half-maximum). The participant's data were filtered in time using a high pass filter (150 s cut-off) and temporal autocorrelations were accounted for by using an AR(1) model [29].

Statistical analysis was performed in SPM2. At the individual participant level the data for each task were modelled with the three conditions (“social”, “gender” and “rest”) each modelled by a boxcar convolved with a canonical haemodynamic response function. Movement was modelled as a covariate of no interest. Contrast images were generated for each participant for the contrast of interest (“social” versus “gender”). Contrast images were entered into second-level random effects analysis using t-tests to examine within-group and between-group effects. Within group regression analysis was performed to determine brain regions in which activity correlated with empathy quotient scores (EQ) and systematising quotient scores (SQ) [1], [49].

Statistical maps were thresholded at P<0.001 uncorrected for within group analysis and P<0.005 for the between group and regression analyses. Regions were considered significant at P<0.05 at the cluster level, corrected for multiple comparisons across the whole brain. Region of interest analysis was conducted to examine the hypothesis that inferior frontal cortex activation differed between ASD group and controls, based on results from the male-female comparison, using an anatomically derived region of interest derived from the aal atlas (WFU PickAtlas) comprising all divisions of the inferior frontal cortex bilaterally [50], [51].
